# The prevalence of hypertension, obesity and dyslipidemia in individuals of over 30 years of age belonging to minorities from the pasture area of Xinjiang

**DOI:** 10.1186/1471-2458-10-91

**Published:** 2010-02-24

**Authors:** Xiao-Guang Yao, Florian Frommlet, Ling Zhou, Feiya Zu, Hong-Mei Wang, Zhi-Tao Yan, Wen-Li Luo, Jing Hong, Xin-Ling Wang, Nan-Fang Li

**Affiliations:** 1 The Center of Diagnosis, Treatment and Research of Hypertension in Xinjiang, Tianchi Road, Urumqi, Xinjiang, China; 2 Institute of Statistics and Decision Support System, University of Vienna, Universitaetsstrasse, A-1010 Vienna, Austria

## Abstract

**Background:**

The prevalence of population-wide hypertension, obesity and dyslipidemia has not been well studied in the pasture area of Xinjiang. The present epidemiological study was performed to determine the prevalence of hypertension, obesity and dyslipidemia in minority populations from the pasture area of Xinjiang and to discuss the potential risk factors for hypertension.

**Methods:**

A population-based, cross-sectional study in the Xinjiang pasture area was performed which included 2251 participants aged over 30 years (90.33% participation rate) of whom 71.26% were Kazaks. Several risk factors were considered: hypertension (defined as systolic or diastolic blood pressure or both of at least 140/90 mmHg measured on one occasion or treatment for hypertension) overweight/obesity (body mass index ≥ 25 kg/m^2^) alcohol intake, smoking/tobacco use and dyslipidemia. Outcomes were prevalence of hypertension, obesity and dyslipidemia and the associated risk factors of hypertension detected by multivariate logistic regression analysis taking into account various metabolic and lifestyle characteristics.

**Results:**

The prevalence of hypertension, overweight/obesity and dyslipidemia in all participants from the pasture area of Xinjiang was 51.9%, 47.9% and 49.2% respectively. Independently, the prevalence and awareness of hypertension was 52.6% and 15.3% among Kazaks (n = 1604), 54.6% and 14.1% among Uygurs (n = 418), 39.5% and 16.1% among Mongolians (n = 81) and 43.9% and 18.2% among non-Xinjiang-born Han immigrants (n = 148). The prevalence of overweight/obesity in Kazaks, Uygurs, Mongolians and Han immigrants was 46.7%, 48.9%, 62.5% and 50.3%, respectively. The prevalence of dyslipidemia in the four ethnic groups mentioned was 53.5%, 34.8%, 49.3% and 47.3%, respectively. The mean blood pressure in all participants was 136/86 mmHg (pre-hypertensive), the mean BMI was 24.7 kg/m^2^. Based on multiple logistic regression analysis, the significant risk factors for hypertension were age [1.07(1.06-1.09), P < 0.0001], overweight/obesity [overweight: 1.61(1.22-2.13), p = 0.0007; obesity: 1.95 (1.33-2.87), p = 0.0007], hypercholesterolemia [1.30(1.15-1.47), p < 0.0001] and an alcohol intake of over 30 g/day [2.22(1.43-3.45), p = 0.0004].

**Conclusions:**

The considerably high prevalence of hypertension, overweight/obesity and dyslipidemia among the minority population aged over 30 from the pasture area of Xinjiang calls for effective preventive measures. Age, increased body mass index, hypercholesterolemia and ≥30 g/d alcohol intake can be counted as risk factors for hypertension, but further genetic or environmental clarification would be desirable to explain the unusually high prevalence of the conditions mentioned above.

## Background

Cardiovascular diseases (CVDs) are the leading cause of death in China [1], as in most industrialized countries. Hypertension, often combined with obesity and dyslipidemia, is one of the four most important predictors of CVDs [2], as well as stroke, being present in more than 70% of all cases [3]. Epidemiological data indicates that the prevalence of hypertension varies greatly between different countries [4] and diverse ethnic populations [5,6].

Kazaks, Uygurs and Mongolians are the major minorities in Xinjiang--the northwest of China. Their main employment is that of herdsmen or peasants in the pasture area. Kazaks, the main nomad ethnic group in the pasture area of Xinjiang, have been reported to have a higher prevalence of hypertension [7], while obesity is common in Uygurs and Mongolians [8]. Liu et al [9] concluded that the significant differences in mean blood pressure and prevalence of hypertension between Han, Kazaks, Uygurs and Tibetan ethnic groups are likely to be caused by different diet-related habits. It is known that alcohol, salty food and meat are traditionally popular among these minorities. This disposition is greatly related to the cold weather in Xinjiang. However, as nomad minorities usually live far from large city centres and move around within the pasture area, large-scale population-based investigations on hypertension, obesity and dyslipidemia are still sparse and the true prevalence among these ethnic groups is therefore not fully known.

The first aim of this study was to assess the prevalence of hypertension, obesity and dyslipidemia in Kazaks, Uygurs and Mongolians aged over 30 from the pasture area of Xinjiang. A secondary aim was to determine if the previously reported high prevalence of hypertension, obesity and dyslipidemia in Xinjiang-born Kazaks could be detected in a large population-based sample and if it could be attributed to metabolic and lifestyle factors.

## Methods

A study of men and women of more than 30 years of age was performed in three counties - Hefeng, Fuhai and Xinyuan, where most of the residents are Kazaks. In the pasture area of Xinjiang between October 1997 and February 1998, all persons older than 30 living in the above three counties were invited to participate in a thorough health screening study. The participants underwent physical examination including measurements of height, weight as well as electrocardiogram. Fasting blood samples were taken for analysis, and a comprehensive questionnaire with validated questions was completed. All participants received preventive individualized cardiovascular lifestyle advice, and participants newly diagnosed for hypertension were referred to primary care or hospitals.

The study was approved by the ethics committee at People's Hospital of Xinjiang Uygur Autonomous Region, and all participants gave their written consent.

### Participants

Nearly all the Kazaks of the province of Xinjiang live in the pasture areas which are mostly located in the north of the region. We used a stratified sampling method to select a representative sample of the general population of Kazaks of this area. Three counties (Hefeng, Fuhai and Xinyuan County) were chosen and, based on the government record of registered residence, one participant was randomly selected from each household.

In this way, a total of 2492 participants, 1051 men and 1441 women, were randomly selected from 28 villages of the three counties and were invited to participate. Those who were younger than 30 years old, 84 males and 156 females, were excluded from the analysis, which is motivated by the higher risk of secondary hypertension in individuals younger than 30 years. Furthermore, one 68-year-old female dwarf with height 93 cm was excluded as well. Finally, 2251 individuals (90.33%) were analyzed, 967 men (42.96%) and 1284 women (57.04%). The proportion of participants from the three counties were: Fuhai (n = 566, 25.14%), Hefeng (n = 1030, 45.76%) and Xinyuan (n = 655, 29.10%). The ethnic distribution of the subjects in the present study were: 1604 Kazaks (71.26%), 418 Uygurs (18.57%) and 81 (3.6%) Mongolians. Additionally, 148 Han immigrants, 6.57% of the total sample, were included in this study. As their original provinces or cultural and geographical regions were unknown, all Han immigrants were considered as one group for the purpose of analysis.

### Hypertension, obesity and dyslipidemia

Blood pressure was measured three times by the same physician from the hospital's hypertension department. After resting for 5 to 10 minutes in a quiet and warm room, blood pressure was measured with a table sphygmomanometer. For the purposes of analysis, the mean of the three measured values was considered. A wider cuff was used if the participant's upper arm circumference was above 32 cm. New hypertension was defined as systolic blood pressure greater than 140 mmHg and/or diastolic blood pressure greater than 90 mmHg. Known hypertension was defined as hypertension or intake of antihypertensive drugs as self-reported in the questionnaire. Hypertension was in any case defined as either new or known according to the standard guidelines [10]. The awareness of hypertension was defined as having been informed of the hypertensive status by a health professional in the previous year.

Body mass index (BMI) was calculated as weight (kg) divided by height squared (m^2^). Obesity was defined as body mass index (BMI) ≥ 30 kg/m^2 ^and overweight as 25 kg/m^2 ^≤ BMI < 30 kg/m^2^.

Blood lipids and lipoproteins were measured on samples obtained after an overnight fast (12 h at least). Total cholesterol (TC), triglyceride (TG), high-density lipoprotein cholesterol (HDL-c), and low-density lipoprotein cholesterol (LDL-c) were measured in EDTA plasma. Hypercholesterolemia was defined as TC >5.7 mmol/L, increased LDL-c as LDL-c >3.6 mmol/L, hypertriglyceridemia as TGs >1.7 mmol/L, and reduced HDL-c as HDL-c <0.9 mmol/L. Dyslipidemia was defined by the presence of one or more than one abnormal serum lipid concentration.

### Lifestyle factors

Smoking habits were coded as current daily smoker, former smoker or lifetime non-smoker. Similarly, the alcohol intake was recorded as current alcohol-intake, former alcohol-intake or no history of alcohol-intake. Furthermore, for participants with current alcohol-intake, the questionnaire had questions concerning present intake of beer, wine, spirits and others. Average daily intake of alcohol was calculated and three categories of drinking groups were defined as follows: no (0 g alcohol/day), <30 g/day and ≥ 30 g/day alcohol intake.

### Statistics

Differences in categorical data among Kazak, Uygur, Mongolian and Han groups were examined by χ^2 ^test and differences in quantitative data by ANOVA, followed by Post-hoc test (Tukey). Logistic regression analysis was performed for exposure dose and risk factors of hypertension. All statistical procedures were performed with SAS 9.1.3 (SAS Institute Inc., Cary, NC, USA). All tests were two tailed and the significance level was set at α = 0.05.

## Results

### General characteristics

Table 1 shows the main blood pressure related variables, metabolic and lifestyle characteristics respectively, of all participants from the pasture area of Xinjiang.

**Table 1:** General characteristics of the whole population

Participants	N = 2251
Male/Female (n)	967/1284
Age (years)	46(38-55)
Systolic BP (mmHg)	136.00 (120.00-160.00)
Diastolic BP (mmHg)	86.00 (77.33-99.33)
PP (mmHg)	50.00 (40.00-64.00)
BMI (kg/m^2^)	24.74 (22.31-27.77)
TC (mmol/L)	4.52 (3.83-5.27)
TG (mmol/L)	0.90 (0.63-1.35)
LDL-c (mmol/L)	2.68 (2.19-3.41)
HDL-c (mmol/L)	1.10 (0.90-1.34)
Hypertension (%)	51.93
Hyperlipidemia (%)	50.79
Hypercholesterolemia (%)	15.38
Hypertriglyceridemia(%)	15.27
Increased LDL-c(%)	20.12
Reduced HDL-c(%)	21.53
Overweight/obesity (%)	47.97
Smoking (n, %)*	
Never	1418 (70.13)
Former	95 (4.7)
Current	509 (25.17)
Alcohol intake (n, %)*	
Never	1450 (74.59)
Former	46 (2.37)
Current	448 (23.05)

For metric variables median and inter quartile range, for categorical variables the absolute and relative frequency for each category are shown. BP: blood pressure; PP: Pulse pressure; BMI: body mass index; TC: total cholesterol; TG: triglyceride; HDL-c: high-density lipoprotein cholesterol; LDL-c: low-density lipoprotein cholesterol.

* 60 (2.67%) individuals under regular antihypertensive treatment were excluded from the assessment of systolic BP, diastolic BP and PP levels.

** Because of 229 missing value for 'smoking' and 307 missing value for 'alcohol intake', the proportions in the table were calculated based on 2022 cases for 'smoking' and 1944 cases for 'alcoholic intake'.

### The awareness of hypertension and the prevalence of hypertension, overweight and dyslipidemia

**Figure 1:**
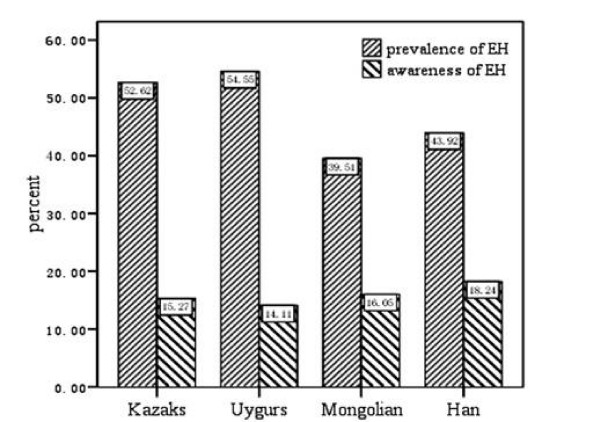
**Prevalence and awareness of hypertension**. Percentage of prevalence and awareness rate of hypertension respectively in Kazaks (1604 cases), Uygurs (418 cases), Mongolians (81 cases) and Han (148 cases) groups from the pasture area of Xinjiang.

The prevalence and awareness of hypertension in all referred participants were 51.93% and 15.28%, respectively. No significant difference between men and women was detected (53.98% vs. 50.39%, χ^2 ^= 2.85, P = 0.09) in the prevalence of hypertension, as well as in the awareness of hypertension (14.89% vs. 15.58%, χ^2 ^= 0.20, P = 0.66). The prevalence and awareness of hypertension in the four ethnic groups are shown in Figure 1. The prevalence of hypertension was highest in Uygur, with 54.55%, and significantly lower in Mongolian, comprising 39.51%. In contrast, the awareness of hypertension was not statistically different between the four ethnic groups (χ^2 ^= 1.48, P = 0.69) and it was quite low in all of them. The prevalence of overweight and obesity in the whole population was 47.96%. There was no difference between men and women (48.5% vs. 47.5%, χ^2 ^= 0.211 P = 0.65) while the prevalence of dyslipidemia was significantly higher in men (52.4% vs. 46.8%, χ^2 ^= 6.41, P = 0.011). The prevalence of overweight and obesity in Mongolians was 62.5%, which was significantly higher than that in Uygurs (62.5% vs. 48.9%, P = 0.003) and that found in Kazaks (62.5% vs. 50.3%, P = 0.005). There seemed to be an even greater difference between Mongolians and the Han population (62.5% vs. 47.3%, P > 0.05) but this was not statistically significant due to the rather small sample size of Han. Similarly, the prevalence of dyslipidemia was 50.79% in the whole study population and varied greatly among the four ethnic groups (F = 15.53, P < 0.0001): it was 53.5% in Kazaks, 49.3% in Mongolians, 47.3% in Han and 34.8% in Uygurs. Pair-wise comparisons of the prevalence of dyslipidemia between the four ethnic groups revealed that only Uygurs had significantly smaller prevalence than Kazaks (P < 0.0001).

### Results of logistic regression

Table 2 shows the odds ratio (OR) of simple and multiple logistic regressions of important factors. Factors associated with hypertension are age, overweight and obesity, plasma TC level and current high alcohol intake (≥30 g/d). Multiple logistic regression showed that, after adjustments for important factors from the simple logistic regression, the ethnic attribution plays an additional role. In particular, the prevalence of hypertension in Mongolians (as well as in Han) was significantly smaller than that found in Kazaks. Within Kazaks, similar factors were detected associating with hypertension as dependent variables: age (OR [95%CI]:1.08 [1.06-1.09], P < 0.0001), total cholesterol (1.38 [1.17-1.64], P = 0.0002), BMI (1.06 [1.02-1.10], P = 0.0048), and more than 30 g alcohol intake per day (4.04 [1.03-15.89], P = 0.0455).

**Table 2:** Simple and multiple logistic regression for covariate factors and their associations to hypertension

factors	Simple logistic		Multiple logistic	
		
	OR(95%CI)	P value	OR(95%CI)	P value
age	1.07(1.06--1.09)	<0.001	1.07(1.06--1.09)	<0.001
male	1(ref)		1(ref)	
female	0.82(0.652--1.02)	0.07	1.15 (0.80--1.65)	0.45
Kazaks	1(ref)		1(ref)	
Uygurs	1.18(0.91--1.54)	0.22	1.03(0.76--1.40)	0.83
Mongolian	0.61(0.31--1.22)	0.16	0.42 (0.20--0.92)	0.03
Han	0.86(0.56--1.31)	0.48	0.62(0.38--1.00)	0.05
normal weight	1(ref)		1(ref)	
overweight	1.73(1.35--2.20)	<0.0001	1.61(1.22--2.13)	0.0007
obesity	2.20(1.55--3.12)	<0.0001	1.95(1.33--2.87)	0.0007
never smoker	1(ref)		1(ref)	
ex-smoker	1.43(0.86--2.40)	0.17	0.90(0.49--1.68)	0.75
daily smoker	1.03(0.80--1.32)	0.83	0.75(0.52--1.08)	0.12
TC	1.35(1.22--1.48)	<0.0001	1.30(1.15--1.47)	<0.0001
TG	1.11(0.95--1.29)	0.19	0.96(0.80--1.14)	0.64
HDL-c	0.96(0.70--1.30)	0.77	0.83(0.58--1.19)	0.43
LDL-c	1.09(0.97--1.24)	0.15	0.94(0.80--1.10)	0.30
never drink	1(ref)		1(ref)	
<30 g/d	1.22(0.82--1.81)	0.33	1.58(0.93--2.66)	0.09
≥30 g/d	1.57(1.17--2.11)	0.003	2.22(1.43--3.45)	0.0004

CI: confidence interval; OR: odds ratios; TC: total cholesterol; TG: triglyceride; HDL-c: high-density lipoprotein cholesterol; LDL-c: low-density lipoprotein cholesterol. Odds ratios plus confidence intervals based on simple and multiple logistic regression. The covariates in the comprehensive model were age, gender, ethnic, obese status (overweight/obesity), cigarette smoking, alcohol intake, plasma total cholesterol, triglyceride, high-density lipoprotein cholesterol and low-density lipoprotein cholesterol.

### Comparison of blood pressure related variables, metabolic and lifestyle characteristics between Kazaks and the other ethnic groups

According to the results showed in Table 1, statistically significant differences were detected in nearly all variables between the four different ethnic groups, except for sex and age. Further comparison between Kazaks and the other three groups was performed (Table 3), where the average SBP, DBP, PP, MAP, PPI and PP/MAP was significantly higher in Kazaks. Though the mean BMI in Kazaks and Uygurs was not statistically different (P = 0.9816), the average plasma lipid profile level was significantly higher in Kazaks than in Uygurs. The mean TC level between Kazaks and the other ethnic groups was not different, but the LDL-c level in Kazaks was significantly the highest. There was some interaction between ethnics and overweight/obesity as determinants of hypertension. The effect of overweight/obesity on both SBP and DBP in Mongolians was significantly smaller than that in Kazaks (P = 0.001 and P = 0.026 respectively). Furthermore, the effect of overweight/obesity on SBP was significantly smaller in Hans than in Kazaks (P < 0.0001).

**Table 3:** The characteristics of the four ethnic groups from the pasture area of Xinjiang

Ethnics	Kazaks	Uygurs	P	Mongolians	P	Han	P
N,%	1604 (71.26)	418 (18.57)		81 (3.6)		148 (6.57)	
Age	47.28 (46.70~ 47.86)	47.36(46.22~ 48.49)	0.9995	48.75(46.85~ 50.65)	0.4695	49.77(47.19~ 52.34)	0.2513
SBP (mmHg)^Δ^	143.01(141.54~ 144.48)	141.52(138.63~ 144.40)	0.8030	134.77(129.92~ 139.63)	0.0080	133.57(127.01~ 140.13)	0.0302
DBP(mmHg)^§^	88.25(87.43~ 89.07)	90.13(88.53~ 91.74)	0.1693	86.70(84.01~ 89.40)	0.7047	82.72(79.07~ 86.36)	0.0195
PP (mmHg)^Δ^	54.76(53.81~ 55.71)	51.38(49.52~ 53.24)	0.0082	48.07(44.95~ 51.20)	0.0004	50.85(46.63~ 55.07)	0.2872
BMI (Kg/m^2^)^§^	25.25(25.04~ 25.46)	25.26(24.85~ 25.67)	1.0000	25.36(24.67~ 26.05)	0.9909	26.79(25.87~ 27.72)	0.0081
TC^§^	4.65(4.58~ 4.72)	4.46(4.33~ 4.59)	0.0622	4.64(4.41~ 4.87)	1.0000	4.36(4.07~ 4.66)	0.2640
TG^Δ^	1.17(1.12~ 1.22)	0.86(0.77~ 0.95)	<.0001	1.42(1.26~ 1.59)	0.0148	1.57(1.37~ 1.78)	0.0010
HDL-c^Δ^	1.17(1.15~ 1.19)	1.01(0.97~ 1.05)	<.0001	2.48(0.97~ 1.10)	0.0005	1.16(1.08~ 1.25)	1.0000
LDL-c^Δ^	2.95(2.90~ 3.00)	2.55(2.47~ 2.64)	<.0001	1.03(2.33~ 2.64)	<.0001	2.49(2.27~ 2.71)	0.0003
Smoking*^Δ^			0.0043		0.0227		0.8057
Never (n,%)	1002(71.47)	266(63.64)		51(86.44)		99(69.23)	
Former(n,%)	65(4.64)	19(4.55)		3(5.08)		8(5.59)	
Current(n,%)	335(23.89)	133(31.82)		5(8.47)		36(25.17)	
Alcohol intake* ^Δ^			<.0001		0.3429		0.0038
Never (n,%)	1030(77.44)	278(66.83)		47(83.93)		95(67.86)	
Former(n,%)	34(2.56)	9(2.16)		0(0.00)		1(0.71)	
Current(n,%)	266(20.00)	129(31.01)		9(16.07)		44(31.43)	

SBP: Systolic blood pressure; DBP: Diastolic blood pressure; PP: Pulse pressure; BMI: body mass index; TC: total cholesterol; TG: triglyceride; HDL-c: high-density lipoprotein cholesterol; LDL-c: low-density lipoprotein cholesterol. Difference between groups was tested with F-test for metric variables and Chi-square test for categorical variables. Significance of the overall test is indicated in the first column by Δ: P < 0.0001 and §: P < 0.05. For metric variables the table provides least square means and 95% confidence intervals, as well as p-values stemming from Tukey post hoc tests (with Kazaks as reference group). For categorical variables frequencies and percentages are listed, as well as p-values from Chi-square tests for the difference between Kazaks and other groups.

* 36 (2.24%)Kazaks, 17(4.07%) Uygurs, 1(1.23%) Mongolian and 6(4.05%) Han individuals under regular antihypertensive treatment were excluded from the analysis of SBP, DBP and PP levels in ethnic groups.

**Due to missing values, proportions in the table were calculated based on 1402 cases for 'smoking' and 1330 cases for 'alcohol intake' in Kazaks, 59 cases for 'smoking' and 56 cases for 'alcohol intake' in Mongolians, 143 cases for 'smoking' and 140 cases for 'alcohol intake' in Han population.

## Discussion

### Prevalence of hypertension, overweight/obesity and dyslipidemia

This epidemiological survey mainly reported considerable prevalence of hypertension (about 52%), overweight/obesity (near 48%) and dyslipidemia (over 50%), combined with low awareness of hypertension (15% only) in the representative sample of the population living in the pasture area of Xinjiang.

The Kazaks, Mongolians and Uygurs recruited in this study are herdsmen or peasants. Their ancestral allocation to the pasture area of Xinjiang goes back for hundreds of years, and they form a relatively homogeneous group with regard to socioeconomic status, occupational and dietary exposures. Few large-scale epidemiological studies have focused on the prevalence of hypertension, overweight/obesity and dyslipidemia among the minorities from the pasture area of Xinjiang in the last decade. Although the large-scale population-based survey in 1995 on the prevalence of hypertension in different ethnic groups in China [11] reported much lower prevalence of the above-mentioned conditions, to our knowledge, most Kazaks and Uygurs involved in the study came from big cities or towns in Xinjiang, while the Mongolians were from the Inner Mongolia Autonomous Region. Therefore, different prevalences were reported in diverse populations and regions, which is a common result of epidemiological investigations. In some small-scale surveys on hypertension in Kazaks, Uygurs and Mongolians of Xinjiang, the results varied greatly. For example, a study including 119 cases [12] showed that the prevalence of hypertension in residential Kazaks of Urumqi was 42.0%. Liu et al [9] data showed the prevalence of Uygurs as 24.3% (264 cases) and Kazaks as 37.9% (99 cases). Data from the Hebukesel pastoral area of Xinjiang demonstrated that the detection rate of hypertension in Kazaks and Mongolians was 55.09% in 2003[13]. In 2005, Wang et al [8] reported in a study of 3732 Mongolians, Kazaks, Uygurs and Hans over 30 years of age in Bortala Prefecture of Xinjiang that the prevalence of overweight and obesity were 36.02% and 27.39% respectively, which was lower than that in our data. Additionally, the low awareness of hypertension displayed in the Guideline for the Prevention and Treatment of Hypertension in China (2005) was confirmed in the present study. Our data calls for the urgent need to develop effective strategies for prevention and treatment of hypertension in Xinjiang.

### Associated risk factors

Our study confirmed the conventional risk factors for hypertension - age, increased BMI, hypercholesterolemia and high alcohol intake (≥30 g/d) - in all participants and specifically in the Kazak population. It is well understood that the aging process affects hypertension [14]. Also positive association between BMI and blood pressure has been well documented [15]. Our study demonstrates that obese individuals, classified by BMI, have significantly larger odds ratio for hypertension than overweight ones. However, we observed significant interaction effects between ethnics and overweight/obesity both for SBP as well as DBP. In particular Mongolians have the highest prevalence in overweight/obesity but the lowest prevalence of hypertension. However, the number of Mongolians included in this study is relatively small, and further research will be necessary to figure out the reasons for this observation.

The results of a 7-year follow-up study on Finnish men [16] suggests that dyslipidemia characteristic of the metabolic syndrome predicts the development of hypertension. Although TG, HDL-C and LDL-c levels were significantly different in our four ethnic groups (Table 3) multiple logistic regression suggested that hypercholesterolemia was positively associated with hypertension in the whole population, not only in Kazaks, which indicates that hypercholesterolemia is a potential determinants of hypertension.

Numerous studies have suggested that excessive alcohol intake causes an increase in blood pressure [17-21]. But in recent years, some researchers claimed that light or moderate alcohol intake might actually benefit hypertension patients [22,23]. Interestingly, in this study, ≥30 g/d alcohol intake was associated with hypertension adjusted for age, sex, smoking and plasma lipid level while the positive association could not be observed for an intake of alcohol <30 g/d. However, only large-scale, prospective and randomized trials might elucidate the actual role of alcohol in hypertension.

Apart from the direct influence of alcohol on blood pressure levels, one might consider that, traditionally among Kazaks, Uygurs and Mongolians in Xinjiang, alcohol consumption is associated with an increased consumption of animal fat or salted food, which could lead to an increase in fibrinogen levels. The male population in particular customarily drinks spirit to deal with the cold weather, as well as having an increased intake of animal fat and salt. Additionally, salted milk tea is enjoyed in large quantities coupled with a low consumption of vegetables, which are scarcely available in the area. Here it should be mentioned that 307 missing values of alcohol intake involved a special alcoholic beverage brewed from horse milk. Over-consumption of this may cause drunkenness, but it is difficult to assess the amount of alcohol within this specific beverage. These missing values may affect the logistic model in the statistical analysis.

Smoking is a well-known risk factor for hypertension and CVD. Association between smoking habits and BP in Japanese men [24], negative dose-effect relationships between the amount of smoking and SBP [25], DBP[26] or both [27] have been reported. On the other hand, some studies have failed to observe a significant dose-effect relationship [28,29]. In this study, no association was found between cigarette smoking and hypertension, for which two potential reasons should be mentioned:

1) hardly any minority women smoke because of ethnical and behavioural restrictions,

2) 229 subjects who failed to report tobacco consumption smoke 'Mohe tobacco' (special tobacco leaves found in Xinjiang) and were considered as missing values for the logistic model.

Some general limitations should be also mentioned. First, the number of minority participants except for Kazaks was too small to allow further analysis. This is a problem when considering the differences between ethnic groups. For example, Han population of Xinjiang mostly consists of immigrants from other provinces, different in cultural background, language, reasons for migration, duration of stay in Xinjiang and age when migrating. Therefore, our conclusions cannot automatically be attributed to all Han immigrants. Second, the study was not primarily designed to compare multiple ethnics and Kazak participants, but the high percentage of residents with different ethnicities in the Xinjiang pasture area made this contingently possible. The data from Mongolians might be biased due to the rather small sample size in this study. Third, blood pressure was measured three times, but only on one occasion, which may overestimate the prevalence of hypertension. Such an over-estimation should, however, be the same in all studied groups, and single-occasion blood pressure measurements are common practice in epidemiological studies [5,30,31]. Finally, we unfortunately did not have data on intake of salt and macronutrient factors that may have an impact on blood pressure [32]. With respect to the high prevalence of hypertension, obesity and dyslipidemia found in Kazaks and Uygurs, there may be a risk of residual confounding by factors we were not able to adjust for, like e.g. the physical activity level of participants. However, keeping these limitations in mind, our study may provide a baseline database and preliminary results for further studies on the association of hypertension and its risk factors with lifestyle in different ethnic groups from the pasture area of Xinjiang.

## Conclusions

In summary, about half of the over 30-year-old minority participants from the pasture area of Xinjiang have hypertension, overweight/obesity and dyslipidemia, respectively, while the awareness of hypertension was as low as 15%. This emphasizes the need for primary preventive measures addressing minorities of the pasture area of Xinjiang, which should not only focus on hypertension but also include its associated risk factors. Further, the genetic and environmental factors and their interaction on hypertension should be considered to clarify the mechanism of hypertension.

## Competing interests

The authors declare that they have no competing interests.

## Authors' contributions

XGY performed the statistical analysis and prepared the manuscript. FF guided the statistical analysis and revised the manuscript. LZ, FZ, HMW, ZTY, WLL, JH, XLW collected the data and reviewed the manuscript. NFL was responsible for the study design and coordination and reviewed the manuscript critically. All authors read and approved the final manuscript.

## Pre-publication history

The pre-publication history for this paper can be accessed here:

http://www.biomedcentral.com/1471-2458/10/91/prepub
